# First Comparison between [18f]-FMISO and [18f]-Faza for Preoperative Pet Imaging of Hypoxia in Lung Cancer

**DOI:** 10.3390/cancers13164101

**Published:** 2021-08-14

**Authors:** Sébastien Thureau, Nicolas Piton, Pierrick Gouel, Romain Modzelewski, Antoine Dujon, Jean-Marc Baste, Jean Melki, Philippe Rinieri, Christophe Peillon, Olivier Rastelli, Justine Lequesne, Sébastien Hapdey, Jean-Christophe Sabourin, Pierre Bohn, Pierre Vera

**Affiliations:** 1 Department of Radiation Oncology, Henri Becquerel Cancer Center and Rouen University Hospital, & QuantIF—LITIS [EA (Equipe d’Accueil) 4108], 76000 Rouen, France; 2 Department of Nuclear Medicine, Henri Becquerel Cancer Center and Rouen University Hospital, & QuantIF—LITIS [EA (Equipe d’Accueil) 4108–FR CNRS 3638], Faculty of Medicine, University of Rouen, 76000 Rouen, France; pierrick.gouel@chb.unicancer.fr (P.G.); romain.modzelewski@chb.unicancer.fr (R.M.); sebastien.hapdey@chb.unicancer.fr (S.H.); pierre.bohn@chb.unicancer.fr (P.B.); pierre.vera@chb.unicancer.fr (P.V.); 3 Department of Pathology, France and Normandie University, UNIROUEN, Inserm U1245, Rouen University Hospital, 76000 Rouen, France; Nicolas.piton@chu-rouen.fr (N.P.); jean-christophe.sabourin@chu-rouen.fr (J.-C.S.); 4 Clinic of Cedre, 76000 Rouen, France; a.dujon@wanadoo.fr; 5 Unit of General and Thoracic Surgery, Rouen University Hospital, 76000 Rouen, France; jean-marc.baste@chu-rouen.fr (J.-M.B.); jean.melki@chu-rouen.fr (J.M.); philippe.rinieri@chu-rouen.fr (P.R.); Christophe.peillon@chu-rouen.fr (C.P.); 6 Unit of Clinic Research, Henri Becquerel Cancer Center, 76000 Rouen, France; Olivier.rastelli@chb.unicancer.fr (O.R.); Justine.lequesne@chb.unicancer.fr (J.L.)

**Keywords:** lung cancer, hypoxia, PET, FMISO, FAZA

## Abstract

**Simple Summary:**

The definition of the tumor hypoxia is important in oncology because this characteristic is linked to a poor prognosis. In this context, we compared two hypoxia tracers, FMISO and FAZA, before surgery for lung cancer. Hypoxia tracers correlate well with each other and FMISO is superior to FAZA in defining the hypoxia volume of lung cancers. However, there is no correlation with immunohistochemical findings (GLUT-1, CAIX, LDH-5, and HIF1-Alpha).

**Abstract:**

Hypoxic areas are typically resistant to treatment. However, the fluorine-18-fluoroazomycin-arabinoside (FAZA) and fluorine 18 misonidazole (FMISO) tracers have never been compared in non small cell lung cancer (NSCLC). This study compares the capability of 18F-FAZA PET/CT with that of 18F-FMISO PET/CT for detecting hypoxic tumour regions in early and locally advanced NSCLC patients. We prospectively evaluated patients who underwent preoperative PET scans before surgery for localised NSCLC (i.e., fluorodeoxyglucose (FDG)-PET, FMISO-PET, and FAZA-PET). The PET data of the three tracers were compared with each other and then compared to immunohistochemical analysis (GLUT-1, CAIX, LDH-5, and HIF1-Alpha) after tumour resection. Overall, 19 patients with a mean age of 68.2 ± 8 years were included. There were 18 lesions with significant uptake (i.e., SUVmax >1.4) for the F-MISO and 17 for FAZA. The mean SUVmax was 3 (±1.4) with a mean volume of 25.8 cc (±25.8) for FMISO and 2.2 (±0.7) with a mean volume of 13.06 cc (±13.76) for FAZA. The SUVmax of F-MISO was greater than that of FAZA (*p* = 0.0003). The SUVmax of F-MISO shows a good correlation with that of FAZA at 0.86 (0.66–0.94). Immunohistochemical results are not correlated to hypoxia PET regardless of the staining. The two tracers show a good correlation with hypoxia, with FMISO being superior to FAZA. FMISO, therefore, remains the reference tracer for defining hypoxic volumes.

## 1. Introduction

Tumour hypoxia is an important influencing factor of tumour fate. It is associated with a poorer prognosis in those who undergo radiation therapy because hypoxia is a major factor of radioresistance [1,2,3,4]. Reference methods (Eppendorf) are invasive and not suitable for deep tumours, such as lung cancers [5]. Positron emission tomography/computed tomography (PET/CT) has been the most studied non-invasive technique for identifying intratumoural hypoxia. In addition, specific tracers have been developed to define the target hypoxic areas in various cancers, including head and neck cancer and lung cancer. PET using the tracers fluorine-18-misonidazole (FMISO) or fluorine-18-fluoroazomycin-arabinoside (FAZA) can be an alternative to identify intratumoural hypoxia [6]. Another indirect marker of hypoxia is fluorine-18-fluorodeoxyglucose (18F-FDG) uptake in PET [3].

Of the hypoxia tracers described, only FMISO and FAZA have been used fto define hypoxic areas. Both tracers are radiolabeled with flourine-18 with a half-life-time of 110 min and belong to the group of 2-nitroimidazoles. Passing the cell membrane is facilitated by their lipophilicity, after which under hypoxic conditions, these compounds are reversibly reduced to highly reactive oxygen radicals [7]. These oxygen radicals bind to intracellular macromolecules at low pO2 values below 5–10 mmHg, and thus, are trapped inside the hypoxic cells. The basis for the application of FDG PET imaging in oncology is the upregulation of glucose transporters (GLUTs) and glycolytic enzymes. It is important to note that tumour hyperglycolysis is driven by the activation of hypoxia-inducible factor-1 (HIF-1). Accordingly, the degree of FDG uptake by tumours might indirectly reflect the level of hypoxia because HIF1-Alpha expression may be observed in non-hypoxic tissues. A recent article recalls the characteristics of hypoxia tracers [8].

FAZA and FMISO have been shown to identify lung cancer patients with poor prognosis, especially before radiotherapy [9,10]. FAZA is a tracer that can clinically detect hypoxia in both primary tumours and lymph node metastasis [10,11,12]. In addition, both FAZA and FMISO can be used for determining the target volume of radiotherapy [11,12]. However, the capability of these tracers to identify target areas compared to that of FDG remains unclear [13]. Moreover, aside from the high cost, the use of these tracers is limited owing to their low contrasts between the tumour and the healthy tissues and consequently the difficulty to define the best method of thresholding [14]. To our best knowledge, no study has compared FAZA and FMISO in NSCLC [15,16]. Thus, this study aimed to compare the capability of 18F-FAZA PET/CT with that of 18F-FMISO PET/CT for detecting hypoxic tumour regions in early and locally advanced NSCLC patients. We also aimed to evaluate the correlation between PET imaging data and histopathological cell markers.

## 2. Materials and Methods

### 2.1. Study Design and Patients

A total of 20 patients with histologically diagnosed NSCLC, eligible for curative surgery (tumour stage ≥T2a without metastasis) were prospectively included (RTEP6; NCT02490696). Each patient underwent both FAZA and FMISO PET before the surgery in no particular order.

### 2.2. FDG/PET Imaging

PET-CT whole-body images were acquired on a GE710 PET-CT device (General Electric, Milwaukee, USA), 60 min (±5 min) after the injection of approximately 3.5 MBq/kg of FDG. PET and CT acquisition parameters were adapted to the patient’s habitus. For patients with a body mass index (BMI) < 30 kg/m^2^, the CT voltage was 100 kV, and the PET acquisition time was 2 min per bed position. Otherwise, the CT voltage was 120 kV, and the PET acquisition time was 2.5 min per bed position. The CT mAs was regulated using the manufacturer’s dose reduction software, yielding a mean effective mAs of 89.1 ± 6.7.

### 2.3. Hypoxia PET Imaging

We injected FAZA or F-MISO at a dose of 4 MBq/kg, and the post-injection delay was 180 ± 10 min. Three bed positions (4 min/bed) were centred on the thorax. CT scan parameters were set to 100 kV and 80 mAs using the manufacturer’s dose reduction software, yielding a mean DLP value of 160.9 ± 44 mGycm.

### 2.4. Qualitative and Quantitative Analysis

PET/CT images using FDG, FAZA, and FMISO were transferred on a Dosisoft workstation (v1.4, Oncoplanet, DosiSoft, Cachan, France). Then the three volumes were first co-registered with a block-matching rigid registration method focusing on the tumour. The physicians were allowed to manually correct obvious misregistration. For each PET, the maximum standardised uptake value (SUVmax), SUVmean, and volume were evaluated at a threshold of 1.4 and 1.5 * SUVmean mediastinum. This assessed whether the FAZA and FMISO areas were similar in volume and in position, using the overlap fraction (OV = V1∩V2/min(V1, V2)), the Dice index (DI) = (2 × (V1∩V2)/(V1 + V2) and the Jaccard index (JI) = (V1∩V2)/(V1UV2).

### 2.5. Parametric Imaging Method

For each patient, a cubic volume of interest (VOI) was selected in the area of the primary tumour on the F-MISO (F-MISO/PET) and FAZA images (FAZA/PET) by an experienced physician. FMISO/PET and FAZA/PET datasets were subtracted, yielding a 3D image of the VOI, with the signal in each voxel i is proportional to the difference in SUV: DIFF(i) = (SUV FAZA–SUV FMISO). Then, the voxels of DIFF were classified into four classes according to the voxel values in both the F-MISO/PET and DIFF datasets. Voxels were classified using a stochastic expectation maximisation algorithm, assuming a Gaussian mixture model for the distribution of voxel values. A parametric dataset was created from DIFF by setting the signal in voxels belonging to Cl3 and Cl4 to zero. The voxels belonging to Cl1 (FMISO superior to FAZA) were coded on a green colour scale, and inversely on a red colour scale for Cl2 (FMISO inferior to FAZA). At the end of the process, the parametric image consisted of one or several clusters of voxels, either red (r) or green (g). The cluster volume (Vr or Vg in cm^3^) was calculated for each cluster.

### 2.6. Immunohistochemistry Analysis

Resected specimens were entirely formalin-fixed, and samples were mapped. For each tumour, one representative section was immunostained, and for each section, one tumour paraffin-embedded block was chosen for immunohistochemical analysis. Mapping, section and block selection were defined jointly by the same pathologist and nuclear physician for all patients based on PET data. Particular attention was paid to obtain the best correlation between immunohistochemistry and PET from the orientation of the surgical specimen to the section. Slides were immunostained using the following primary antibodies as hypoxia markers: Anti-HIF1-Alpha (1:500; ab8366; Abcam^®^, Cambridge, UK), anti-GLUT-1 (1:250), anti-CAIX (1:50), and anti-LDH-5 (1:1000). Deparaffinisation, antigen retrieval, and immunostaining were performed using a Benchmark ULTRA^®^ device (Ventana-Roche^®^; Oro Valley, Arizona, USA). Slides were heated to 72 °C, incubated for 30 min with the antibody, and then rinsed in buffer solution. Primary antibody detection was performed using Ultraview DAB solution (Roche-Ventana^®^; Oro Valley, Arizona, USA). After rinsing, a drop of haematoxylin solution was applied within 4 min for counter-staining, before rinsing again. Finally, the slides were mounted with a glass coverslip.

### 2.7. Staining Assessment

Immunostained slides were observed using a dual-headed microscope (Leica DM 2000, Wetzlar, Deutscland) by pathologists who were blinded to the results of the PET analysis. Only malignant cells were scored. HIF1-Alpha, GLUT-1, CAIX, and LDH5 expression was evaluated according to a method described by Allred et al. [17]. This analysis was independently performed by three different pathologists, and the mean score was then used for the subsequent analysis.

## 3. Statistical Analysis

SUV and immunohistochemical scores were described using mean and SD. SUVmax FMISO and FAZA were compared with a paired t-test and their correlation using the Pearson correlation coefficient. *p*-values < 0.05 were considered statistically significant (bilateral test). Finally, volume concordance was estimated by computing overlap fraction, DI, and JI. The association between PET endpoints and immunohistochemical scores was explored by computing non-parametric Spearman correlation coefficients. All analyses were performed using MedCalc Statistical Software (MedCalc Software bvba, Ostend, Belgium). An alpha risk level of 5% was retained for each statistical test.

## 4. Results

### 4.1. Patient Characteristics

Initially, 23 patients were screened, but two patients could not undergo the two hypoxia PET imaging before the surgery and two had histologies other than NSCLC. The study was closed after the pre-inclusion of the 23 patients. Thus, 19 patients (4 women, 15 men) with a mean age of 68.2 ± 8.2 years were included in the study. The histological subtypes were adenocarcinoma in nine patients and squamous cell carcinoma in 10 patients. One patient had stage IA3 disease; four patients, stage IB; one patient, stage IIA; three patients, stage IIB; seven patients, stage IIIA; and one patient, stage IIIB (TNM 8th edition). The patients’ characteristics are shown in Table 1. The mean interval between the two PET modalities was 2.1 days (±1.6), and between the last PET and the surgery was 3.1 days (±2.8) (Table 1).

### 4.2. PET Results

**Figure 1:**
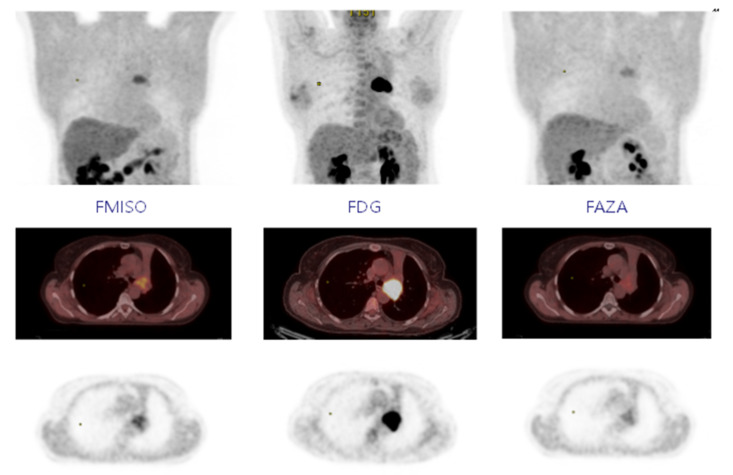
Fixing FMISO, FDG, and FAZA on MIP, axial PET-CT, and axial PET.

For FDG PET, the SUVmax was 12.4 (±5.5), and the volume at a threshold of 40% for SUVmax was 23.2 cc (±19.2). In FMISO PET, 18 of the 19 lesions were considered hypoxic with a SUVmax greater than 1.4. Figure 1 shows the MIP (Maximum intensity projection), axial PET/CT slice and axial PET slice for FMISO, FDG and FAZA, respectively; the MIP is used to represent the tumour-to-background ratio. The mean SUVmax was 3 (±1.4) with a mean volume of 25.8 cc (±25.8) for a threshold at 1.4. The SUVmean mediastinum was 1.31 (±0.13). At a threshold of 1.5* SUVmean mediastinum, 15 lesions were hypoxic with a mean volume of 9.04 cc (±13.54). In FAZA PET, 17 lesions were considered hypoxic at a threshold of 1.4 (same lesions in FMISO PET). The mean SUVmax was 2.2 (±0.7) with a mean volume of 13.06 cc (±13.76). The SUVmean mediastinum was 1.15 (±0.15), and 15 lesions were considered hypoxic at a threshold of 1.5* SUVmean mediastinum for a mean volume of 4.97 cc (±5.53). However, five lesions had a volume <1 cc) (Table 1).

**Figure 2:**
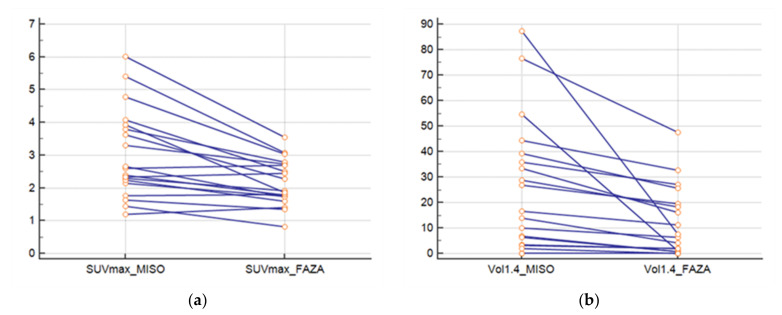
(**a**) SUVmax of lesions for FAZA and FMISO and (**b**) volumes of lesions with FAZA and FMISO (threshold at 1.4).

The SUVmax FMISO was greater than SUVmax FAZA (*p* = 0.0003) (Figure 2a). The ratio between the SUVmax FMISO and FAZA of the lesion was 1.39 (±0.33), and the ratio for the SUVmean mediastinum (FMISO/FAZA) was 1.14 (±0.12). At a threshold of 1.4; the FMISO volume was greater than the FAZA volume (*p* = 0.004) (Figure 2b), and the ratio of the volume was 6.41 (±13.61). For a threshold at 1.5* SUVmean mediastinum, the ratio was 4.84 (±6.43).

**Figure 3:**
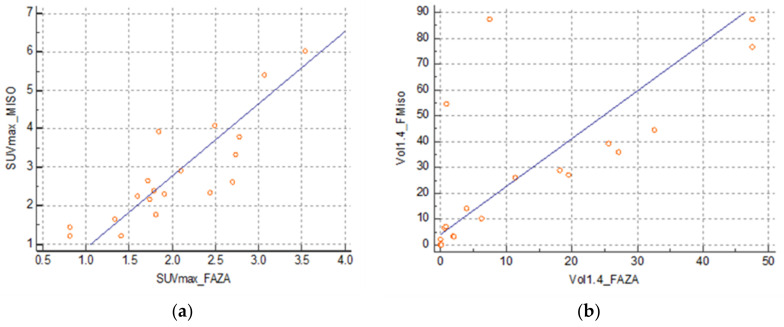
(**a**) Correlation between SUVmax FMISO and FAZA and (**b**) correlation between volumes FMISO and FAZA with a threshold of 1.4.

There was a strong correlation between the SUVmax FMISO and SUVmax FAZA at 0.86 (0.66–0.94) (SUVmax FMISO = −0.5483 + 1.6571 SUVmax FAZA) (Figure 3a) and between the volume at a threshold of 1.4 at 0.63 (0.24–0.84) (FMISO volume = 11.9042 + 1.1869 FAZA volume) (Figure 3b). However, there was no correlation between SUVmax FDG and SUVmax FMISO: 0.32 (−0.16–0.68) and between SUVmax FDG and SUVmax FAZA: 0.44 (−0.02–0.74).

### 4.3. Overlap Fraction, Parametric Results, and Immunohistochemistry Results

With respect to overlap fraction, we confirmed a strong correlation of the two tracers with a mean OV at 0.89 (±0.18). The DI and the Jaccard Index were low with, respectively, 0.53 (±0.26) and 0.4 (±0.24).

**Figure 4:**
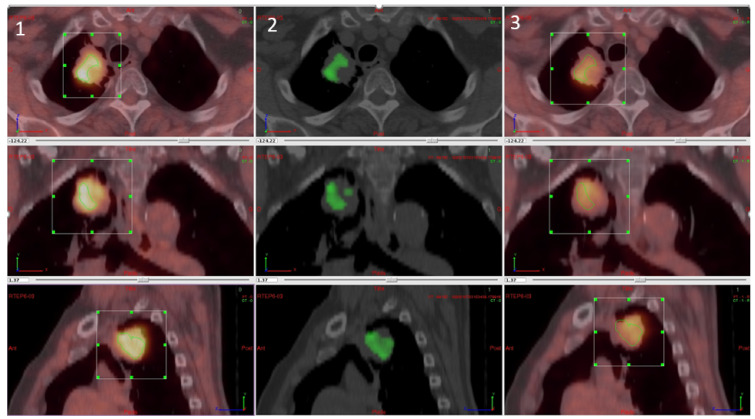
Differential analysis between FMISO and FAZA with (**1**) FMISO PET, (**2**) differential fixation, and (**3**) FAZA PET.

For the parametric results, the differential volume between FMISO and FAZA was determined for all tumours regardless of tumour uptake (inferior and superior to SUVmax at 1.4). There was a significant difference between the two tracers in 17 lesions. Of these, FMISO was superior to FAZA in 15 lesions with respect to fixation, while FAZA was superior in the other two lesions. The difference, however, was not significant. In five lesions, there was more than one differential volume (2–4; 3 cases in favour of FMISO and two cases in favour of FAZA). The mean differential volume was 8.09 cc (±7.19). Figure 4 shows a patient with a significative difference of fixation between FMISO and FAZA in favour FMISO.

**Figure 5:**
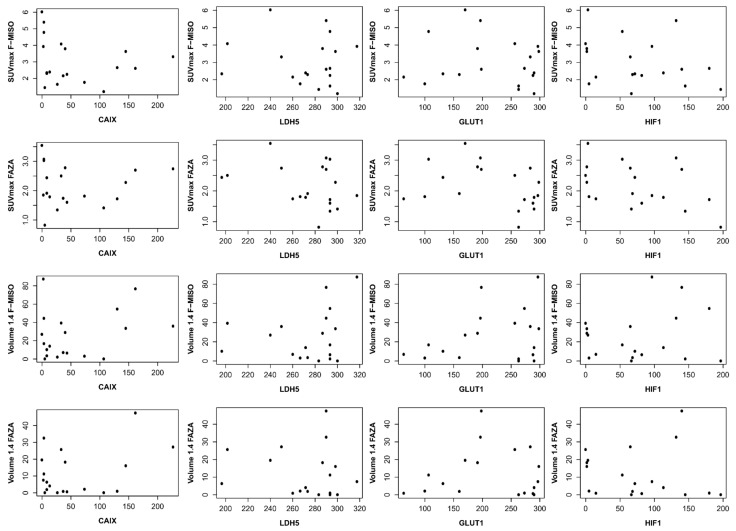
Correlation between immunohistochemical and hypoxia PET. On the ordinate, it is the SUV max for the first two lines and the volume in cc for the last two lines. On the abscissa, the immunohistochemistry score (score described by Allred et al. [17]).

With respect to CAIX immunohistochemistry, the mean score was 56.2 (±64.0); for HIF1, the mean score was 79.6 (±53.3). For LDH5, the mean score was 263.6 (±60.4), and for GLUT1, 216.9 (±767) (Table 2). We found no correlation between the different immunohistochemical analyses and with hypoxia PET. Each hypoxia tracer (FMISO and FAZA) for volumes and intensity was compared with immunohistochemical scores (HIF1-Alpha, GLUT-1, CAIX, and LDH5) (Table 3 and Figure 5).

## 5. Discussion

The capability of F-MISO and FAZA as tracers for hypoxic areas in NSCLC has never been compared. In the present study, we analysed the hypoxic tumour subvolumes in NSCLC using FMISO, FAZA, FDG PET/CT, and immunohistochemistry. To our best knowledge, this is the first study to measure hypoxic volumes in lung cancer using FMISO and FAZA as tracers of hypoxia with a nitroimidazole core. The three PET modalities were administered within short intervals (2 days between FMISO and FAZA) and before surgery (3 days). We found no correlation of FDG with FMISO and FAZA, but a strong correlation between FMISO and FAZA.

The usefulness of hypoxia PET has been mainly evaluated in inoperable patients or patients with cancers in other sites (e.g., the head and neck, prostate, and brain) [11,18,19,20,21,22,23]. With hypoxia being a primary factor of radioresistance, FMISO has been reported to identify patients with poor prognosis before radiotherapy [9,12,24]. A recent meta-analysis confirms the prognostic character of hypoxia PET in head and neck cancers before radiotherapy with either FMISO or FAZA with similar relative thresholds [25]. Several previous studies in NSCLC proposed to increase the dose on the hypoxic volume [RTOG1106, 12]. However, while both FMISO and FAZA as hypoxia tracers were compared to FDG, there is no direct comparison between FMISO and FAZA [13]. In this study, we highlight the strong correlation between the two hypoxic tracers, both for the SUVmax and for the volume. Despite exams that were conducted at 2-day intervals, similar data were found for the two tracers, confirming their reliability. Meanwhile, the weak correlation between FDG and these two tracers confirms that they have different capabilities. FMISO was superior to FAZA as evidenced by the better uptake in absolute value (SUVmax) or in relative value (SUVmax / SUVmean of the mediastinum) in 16 of the 19 patients. This shows that FMISO should be the preferred tracer of hypoxic tissues for NSCLC. The superiority of one tracer over the other has not been demonstrated and varies according to the studies, but the number of patients is always limited, making it difficult to conclude. So, the effectiveness of hypoxia-specific PET tracers might be tumour-dependent.

The method of segmentation to define the hypoxic volume remains to be defined because the threshold defined by our team in previous work [14] lacked specificity (with FMISO, 18 lesions had a fixation higher than 1.4). The stability of fixation of the FMISO in the mediastinum confirms the interest of the relative threshold used by the RTOG. Other healthy tissues can be used because we obtained the same results for the muscles.

The lack of correlation between FDG PET and hypoxia PET confirms that these tracers provide different information and are independent, but complementary. Overall, they might be used for prognostication. Many studies found a regional tumour heterogeneity defined by PET and poor spatial overlap between FMISO uptake and FDG uptake. Tumours with high FDG uptake are more likely to have high cellularity and more rapid proliferation leading to a higher potential for hypoxia [1,26].

To our best knowledge, this first study comparing two hypoxia tracers and immunohistochemical analyses for lung cancer before surgery. Other studies were carried out using a single tracer and often on more limited populations with other tumour locations, such as the head and neck, prostate, or brain [23,27,28,29]. Results on the correlation between immunohistochemistry and hypoxia PET are conflicting [30]. Beckaet et al., in a study on glioblastoma, found a strong correlation with FMISO fixation and the immunohistochemistry markers HIF1alpha and CAIX [23]. Only one study compared FAZA with immunohistochemistry in NSCLC and found no correlation, which could probably be due to the small sample size. One hypothesis is that hypoxia PET can show an acute phenomenon of hypoxia; however, our data showed a strong correlation between both tracers of hypoxia despite that hypoxic PET data were acquired over two days. This result confirmed the usefulness of these PET tracers to define areas of tumour hypoxia. In glioblastoma, a good correlation has been found between FMISO and immunohistochemistry, although surgical methods do not allow a good anatomical correlation between the two methods. Kawai et al. reported that preoperative FMISO uptake in tumours was significantly correlated with the expression of vascular endothelial growth factors for newly diagnosed tumours. However, HIF1-Alpha was not correlated to FMISO [31]. The other hypothesis would be that PET shows a global phenomenon of hypoxia when immunohistochemistry highlights specific processes of this mechanism. Indeed, Hypoxia induces the synthesis of multiple proteins, which can be considered biomarkers regarding this biological condition, and the expression of these proteins can be quantified by immunohistochemistry. Hypoxia-inducible factor (HIF) plays a major role in oxygen detection and adaptation to hypoxia. For example, HIF promotes the expression of glucose transporter, for example, glucose transporter 1 (GLUT-1), enhancing aerobic glycolysis and increasing the transport of glucose [32,33]. In parallel, especially in cancer cells, the activation of the HIF pathway dramatically upregulates the transcription of Carbonic Anhydrase IX (CAIX) [34]. Another biomarker for hypoxia is Lactate Dehydrogenase 5 (LDH5), a crucial enzyme involved in the transformation of pyruvate to lactate for ATP production under anaerobic conditions [35]. LDH5 is composed of subunits encoded by the Lactate Dehydrogenase A (LDHA) gene, whose expression is triggered by HIF [36]. In addition, FDG uptake can be correlated with tumour characteristics, such as programmed death-ligand 1 expression [37,38], and is also an independent prognostic factor. Further studies are needed for the correlation between images and immunohistochemistry, but the accuracy of the methodology will be difficult in humans.

## 6. Conclusions

In conclusion, the results of this study confirm a strong correlation between fluorine-18-misonidazole (FMISO) and fluorine-18-fluoroazomycin-arabinoside (FAZA), indicating that they can both be used to detect hypoxic tumour areas via positron emission tomography/computed tomography (PET/CT) and define hypoxic volumes for radiotherapy. In particular, FMISO showed the superior capability to FAZA. Meanwhile, the lack of correlation with immunohistochemistry findings highlights the need for a definition of new methods to compare imaging and pathology. Larger studies are needed for more conclusive results regarding the most optimal PET tracer for the detection of hypoxic subvolumes in NSCLC.

## Figures and Tables

**Table 1:** Patient characteristics.

Patient	Sex	Age	Stade	Pathology	Delay		SUVmax		
					FAZA/FMISO	PET/Surgery	FDG	FMISO	FAZA
1	M	78	T2bN1	SCC	6	3	12.12	2.39	1.79
2	M	57	T2aN0	ADK	1	1	5.34	2.15	1.74
3	W	64	T2aN0	ADK	1	1	11.33	6.02	3.54
4	M	76	T3N0	SCC	2	2	26.97	3.31	2.74
5	W	65	T4N1	SCC	1	5	21.56	3.79	2.78
6	M	65	T4N0	SCC	1	1	10.03	2.6	2.7
7	M	67	T3N1	SCC	1	1	7.3	2.65	1.72
8	M	75	T2aN0	ADK	6	3	8.72	2.3	1.91
9	M	59	T2aN2	SCC	1	9	15.93	2.24	1.6
10	M	66	T2bN0	SCC	1	6	15.21	4.08	2.5
11	M	69	T2bN2	ADK	1	10	11.67	5.4	3.07
12	W	77	T2bN2	ADK	1	2	9.92	2.46	2.29
13	M	75	T1cN1	SCC	2	1	5.32	1.2	1.75
14	M	63	T2aN0	ADK	1	1	15.45	1.77	1.81
15	W	51	T3N2	ADK	3	3	7.96	1.64	1.34
16	M	65	T1cN0	ADK	3	4	10.38	1.44	0.82
17	M	68	T4N0	SCC	3	1	9.5	3.92	1.85
18	M	83	T3N0	SCC	3	1	12.95	3.63	2.28
19	M	72	T2aN0	ADK	3	2	15.60	4.78	3.03
Mean		68.2			2.1	3.1	12.4	3.0	2.2
SE		8.2			1.6	2.8	5.5	1.4	0.7

M, man, W, woman, ADK, adenocarcinoma, SCC, squamous cell carcinoma.

**Table 2:** Immunohistochemical analysis.

Patient	GLUT1	(SE)	LDH5	(SE)	HIF1	(SE)	CAIX	(SE)
1	290.0	(17.3)	271.7	(40.7)	113.3	(28.9)	13.3	(2.9)
2	63.3	(25.2)	260.0	(69.3)	15.0	(15.0)	36.7	(5.8)
3	170.0	(11.5)	250.0	(17.3)	3.3	(2.9)	0.0	(0.0)
4	283.3	(28.9)	250.0	(43.6)	65.0	(15.0)	226.7	(68.1)
5	191.7	(45.4)	286.7	(11.5)	1.7	(2.9)	40.0	(36.1)
6	198.3	(31.7)	290.	(17.3)	140.0	(32.8)	161.7	(59.6)
7	273.3	(15.3)	293.3	(11.5)	180.0	(55.7)	130.0	(10.0)
8	160.0	(34.6)	273.3	(25.2)	68.3	(46.5)	8.3	(10.4)
9	288.3	(20.2)	293.3	(11.5)	81.7	(46.5)	43.3	(2.9)
10	256.7	(15.3)	201.7	(122.1)	0.0	(0.0)	33.3	(16.1)
11	196.7	(51.3)	290.0	(10.0)	131.7	(86.1)	3.3	(5.8)
12	131.7	(27.5)	196.7	(45.1)	71.7	(43.1)	8.3	(7.6)
13	290.0	(10.0)	300.0	(0.0)	66.67	(55.1)	106.7	(28.9)
14	100.0	(173.2)	266.7	(57.7)	5.0	(8.7)	73.3	(66.6)
15	263.3	(47.3)	293.3	(11.5)	145.0	(48.2)	26.7	(20.8)
16	263.3	(15.3)	283.3	(20.8)	196.7	(73.7)	5.0	(5.0)
17	296.7	(5.8)	317.5	(32.5)	96.7	(25.2)	2.5	(2.5)
18	298.3	(2.9)	298.3	(2.9)	1.7	(2.9)	145.0	(18.0)
19	106.7	(57.7)	293.3	(11.5)	53.3	(28.4)	3.3	(2.9)
Mean	216.9	(33.5)	263.6	(29.6)	79.6	(32.5)	56.2	(19.5)
SE	76.7		60.4		53.3		64.0	

**Table 3:** Correlation between immunohistochemical and hypoxia PET.

F	CAIX		LDH5		GLUT1		HIF1	
	ρ	*p*-value	ρ	*p*-value	ρ	*p*-value	ρ	*p*-value
SUVmax FMISO	−0.32	0.17	−0.08	0.74	−0.04	0.87	−0.35	0.14
SUVmax FAZA	−0.21	0.40	−0.32	0.18	−0.35	0.15	−0.47	0.04
Volume 1.4 FMISO	0.06	0.79	0.07	0.77	0.19	0.43	−0.03	0.89
Volume 1.4 FAZA	0.03	0.89	−0.26	0.28	−0.09	0.71	−0.36	0.13

ρ, correlation coefficient.

## Data Availability

All data from the study are available at the Henri Becquerel Center (URC).

## Abbreviations

BMI	body mass index
DI	Dice index
FAZA	fluorine-18-fluoroazomycin-arabinoside
FMISO	fluorine-18-misonidazole
FDG	fluorodeoxyglucose
JI	Jaccard index
SUVmax	maximum standardised uptake value
NSCLC	non-small cell lung cancer
PET/CT	positron emission tomography/computed tomography
VOI	volume of interest
